# Gastrointestinal absorption of pimozide is enhanced by inhibition of P-glycoprotein

**DOI:** 10.1371/journal.pone.0232438

**Published:** 2020-10-29

**Authors:** Hiroki Morishita, Kozue Okawa, Misaki Ishii, Kenta Mizoi, Masa-aki Ito, Hiroshi Arakawa, Kentaro Yano, Takuo Ogihara

**Affiliations:** 1 Laboratory of Clinical Pharmacokinetics, Graduate School of Pharmaceutical Sciences, Takasaki University of Health and Welfare, Nakaorui-machi, Takasaki, Gunma, Japan; 2 Department of Pharmacy, Saiseikai Maebashi Hospital, Kamishinden-machi, Maebashi, Gunma, Japan; 3 Laboratory of Biopharmaceutics, Faculty of Pharmacy, Takasaki University of Health and Welfare, Nakaorui-machi, Takasaki, Gunma, Japan; 4 Laboratory of Pharmacology, Faculty of Pharmacy, Takasaki University of Health and Welfare, Nakaorui-machi, Takasaki, Gunma, Japan; 5 Faculty of Pharmacy, Institute of Medical, Pharmaceutical and Health Sciences, Kanazawa University, Kakuma-machi, Kanazawa, Japan; Hungarian Academy of Sciences, HUNGARY

## Abstract

Drug-drug interaction was suggested to have played a role in the recent death due to cardiac arrest of a patient taking pimozide, sertraline and aripiprazole antipsychotic/antidepressant combination therapy. Here, we investigated the possible involvement of P-glycoprotein (P-gp)-mediated interaction among these drugs, using in vitro methods. ATPase assay confirmed that pimozide is a P-gp substrate, and might act as a P-gp inhibitor at higher concentrations. The maximum transport rate (*J*_max_) and half-saturation concentration (*K*_t_) for the carrier-mediated transport estimated by means of pimozide efflux assay using P-gp-overexpressing LLC-GA5-CoL150 cells were 84.9 ± 8.9 pmol/min/mg protein, and 10.6 ± 4.7 μM, respectively. These results indicate that pimozide is a good P-gp substrate, and it appears to have the potential to cause drug-drug interactions in the digestive tract at clinically relevant gastrointestinal concentrations. Moreover, sertraline or aripiprazole significantly decreased the efflux ratio of pimozide in LLC-GA5-CoL150 cells. Transport studies using Caco-2 cell monolayers were consistent with the results in LLC-GA5-CoL150 cells, and indicate that P-gp-mediated drug-drug interaction may occur in the gastrointestinal tract. Thus, P-gp inhibition by sertraline and/or aripiprazole may increase the gastrointestinal permeability of co-administered pimozide, resulting in an increased blood concentration of pimozide, which is known to be associated with an increased risk of QT prolongation, a life-threatening side effect.

## Introduction

Pimozide is an antipsychotic used to treat schizophrenic and pediatric autistic disorders. However, one of its major side effects is QT prolongation [1], because it is a strong antagonist of the alpha subunit of a potassium ion channel (hERG) [2] and this action causes a significant prolongation of QT intervals [3]. Several antidepressants are restricted for use in combination with pimozide, because of the increased risk of this side effect. For example, the combination of pimozide and sertraline is contraindicated because it leads to an increased blood concentration of pimozide [4], thus increasing the risk of QT prolongation. Nevertheless, a case has been reported in which a male child administered pimozide together with sertraline and aripiprazole died due to cardiac arrest in Japan [5]. There are several reasons why the blood concentration of pimozide may be elevated by sertraline and/or aripiprazole, but the precise mechanism of this drug-drug interaction is unclear. Since the bioavailability of pimozide is 50% and almost no unchanged drug is excreted in urine, it is considered to be a hepatically metabolized drug [6]. One explanation could be decreased metabolism of pimozide, which is a substrate of cytochrome (CYP) 3A4, 2D6, and 1A2. It has been reported that elevated blood levels of pimozide can occur when it is administered in combination with CYP-inhibitory drugs, resulting in side effects [7]. Aripiprazole is a substrate of CYP3A4 and 2D6 [6], while sertraline is a substrate of 2C19, 2C9, 2B6 and 3A4. Sertraline is also reported to be a mild to moderate inhibitor of CYP2D6 and a weak inhibitor of CYP3A4 [8]. Therefore, it had been suspected that interaction among these drugs would be due to inhibition of CYP-mediated pimozide metabolism by sertraline and/or aripiprazole [9]. On the other hand, crossover clinical studies by Alderman showed that the Cmax and AUC of pimozide were increased by 35% and 37%, respectively, when the drug was used in combination with sertraline, without any significant change of the blood half-life [4]. This indicates that sertraline does not affect pimozide elimination (hepatic metabolism or excretion), but increases the rate and amount of oral absorption. An increase in absorption rate and/or amount suggests inhibition of efflux transporters and/or metabolic enzymes in the gastrointestinal tract. Among the metabolic enzymes expressed in the gastrointestinal tract, only CYP3A4 is involved in the metabolism of pimozide. However, a significant role of CYP3A4 seems unlikely, because if sertraline inhibits CYP3A4 in the gastrointestinal tract, CYP3A4-mediated interaction in the liver should also affect the pimozide elimination phase, but the observed blood concentration levels of pimozide indicate that this was not the case.

P-Glycoprotein (P-gp) is a member of the ATP-binding cassette superfamily, and is mainly localized at the intestine, blood-brain barrier, adrenal gland, enterocytes, hepatocytes, placenta and renal proximal tubules in humans [10]. P-gp is responsible for the efflux of many xenobiotics and plays major roles in drug absorption, distribution and excretion. In the intestine, P-gp mediates the efflux of its substrates, restricting the absorption of many xenobiotics, including drugs [11–14]. Recent studies show that sertraline, aripiprazole, and several of their metabolites have a P-gp-inhibitory effect [15–17], although other in vivo studies have found that sertraline did not alter digoxin or fexofenadine pharmacokinetics [18,19].

Pimozide has been described as a substrate of P-gp [20], and as an inhibitor of P-gp [21,22], but those reports did not provide definitive data. If pimozide is a substrate of P-gp, its absorption may be increased when it is used in combination with P-gp inhibitors.

In this study we examined whether pimozide is a substrate of P-gp and evaluated whether gastrointestinal absorption of this drug may be increased as a result of P-gp inhibition by sertraline and/or aripiprazole by means of in vitro experiments.

## Materials and methods

### Chemicals

Pimozide (#P1793-500MG) was purchased from Santa Cruz Biotechnology (Dallas, TX). Sertraline hydrochloride (#193–16191), aripiprazole (#017–23831) and verapamil hydrochloride (#222–00781) were purchased from Wako Pure Chemical Industries (Osaka, Japan). All other reagents were commercial products of reagent grade.

### ATPase assay

The SB-MDR1 PREDEASYTM ATPase Kit, including P-gp-expressing membrane vesicles, was purchased from SOLVO Biotechnology (distributed by KAC Co., Ltd., Kyoto, Japan). This assay kit measures the interaction of test drugs with P-gp. ATPase assay was conducted according to the manufacturer’s instructions and a previous report [23]. Inorganic phosphate liberated by ATP hydrolysis was detected by colorimetric reaction. The optical density (OD) was measured at 590 nm with a microplate reader, Sunrise^™^ Rainbow (TECAN, Kanagawa, Japan).

### Cell culture

LLC-GA5-CoL150 cells (RRID:CVCL_U207), a derivative of kidney epithelial cell line LLC-PK1 expressing human P-gp on the apical membrane, were obtained from Riken Cell Bank (Tsukuba, Japan). They were cultured, passaged and grown as described previously [24] in Medium 199 supplemented with 10% fetal bovine serum, 100 U/mL penicillin, 100 μg/mL streptomycin and 150 ng/mL colchicine, at 37°C in an atmosphere of 5% CO_2_ in air. The cells were seeded on 12-well Transwell^®^ collagen-coated filter membrane inserts (#3494, Costar, Bedford, MA, USA) at a density of 2.5 × 10^5^ cells/cm^2^ for transport studies [24], and seeded on 24-well cell culture plates (Corning, NY, USA) at a density of 1.5 × 10^5^cells/cm^2^ for efflux assays [25]. The culture medium was replaced with fresh medium every second or third day. Cells were grown for 7 days and used for experiments. One day prior to experiments, the medium was changed to Medium 199 without colchicine. Transepithelial electrical resistance (TEER) was measured using a Millicell-ERS resistance system (Millipore, Bedford, MA, USA). Cell monolayers with TEER values of 300 to 600 Ω·cm^2^ were used for transport studies.

Caco-2 cells (RRID:CVCL_0025), a human colon epithelial cancer cell line frequently used as an in vitro intestinal model, were purchased from the American Type Culture Collection (Rockville, MD, USA). The cells were cultured, passaged and grown as described previously [26] in Dulbecco’s modified Eagle’s medium supplemented with 10% fetal bovine serum, 100 units/ml penicillin, 0.1 mg/ml streptomycin, and 1.0% nonessential amino acids, at 37°C in an atmosphere of 5% CO_2_ in air. The cells were seeded on 12-well Transwell^®^ polycarbonate filter membrane inserts (#3402, Costar, Bedford, MA, USA) at a density of 6 ×10^4^ cells/cm^2^. The culture medium was replaced with fresh medium every second or third day and Caco-2 cell monolayers grown for 21 days were used for the transport studies. Cell monolayers used for transport studies had TEER values of 800 to 1000 Ω·cm^2^.

### Efflux rate assay

Efflux assay with LLC-GA5-CoL150 cells was performed as previously described [23], with some modifications. Cells seeded on 24-well cell culture plates were washed twice with ice-cold Dulbecco’s phosphate-buffered saline (-) (D-PBS(-)), then ice-cold Opti-MEM^®^ containing 0.01 to 100 μM pimozide was added. The cells were incubated for 30 min at 4°C, washed twice with ice-cold D-PBS(-), and further incubated in Opti-MEM^®^ for 10 min at 37°C. The cells were then washed three times with ice-cold D-PBS(-), dried, and lysed by adding 200 μL of 0.1 N NaOH solution. Cell lysates were used for protein determination and measurement of pimozide concentration. Initial uptake of pimozide was evaluated using cells incubated with Opti-MEM containing pimozide for 30 min at 4°C. The efflux rate was calculated according to the following formula.

$$\begin{array}{l} \text{Efflux}\;\text{rate}\;(\text{pmol/min/mg}\;\text{protein})\;=\;\{\text{Initial}\;\text{uptake}\;\text{of}\;\text{pimozide}\;(\text{pmol/mg}\;\text{protein})-\\ \text{Residual}\;\text{amount}\;\text{of}\;\text{pimozide}\;\text{after}\;10\;\text{min}\;\text{incubation}\;(\text{pmol/mg}\;\text{protein})\}/10\;(\text{min}) \end{array}$$

Protein was determined colorimetrically using DCTM Protein Assay (BIO-RAD, Hercules, CA), based on absorbance measurement at 700 nm with a microplate reader, Sunrise Rainbow RC (Tecan, Kanagawa, Japan). An aliquot of cell lysate was mixed with 300 μL of ethyl acetate for 10 min in the cold, then 100 μL of the organic phase was taken and evaporated. The residue was dissolved in 300 μL of mobile phase. All samples were applied to MultiScreen^®^ Solvent Filter Plates 0.45 μm Low-Binding Hydrophilic PTFE (Merck, Ireland) and centrifuged at 3,500 rpm for 20 min at 4°C. Filtered samples were collected in a 96-well microplate (AS ONE, Tokyo, Japan), and pimozide concentration was determined by triple quadrupole liquid chromatography mass spectrometry (LC-MS/MS) as described below.

To estimate the kinetic parameters of carrier-mediated transport in the efflux assays, the transport rate (*J*) was fitted to the following Eq (1), containing saturable and nonsaturable-linear terms, by using the nonlinear least-squares regression analysis program, MULTI, as previously reported [27].

$$J\;=\;J_{max}\;\times \;[S]\;/(K_{t}\;+\;[S])\;+\;k_{d}\;\times \;[S]$$ (1)

Where *J*_max_ is the maximum transport rate for the carrier-mediated transport, [*S*] is the substrate concentration, *K*_t_ is the half-saturation concentration, and *k*_d_ is the first-order rate constant.

### Bidirectional assay

In the transport studies with LLC-GA5-CoL150 cell monolayers, the transport medium on both sides consisted of Hanks’ balanced salt solution (HBSS) with 10 mM 2-[4-(2-hydroxyethyl)-1- piperazinyl]ethanesulfonic acid (HEPES) (pH 7.4). LLC-GA5-CoL150 cell monolayers were preincubated with the transport medium for 20 min at 37°C. Transport experiments were initiated by adding the transport medium containing pimozide (10 μM) to the donor side. The receiver side contained transport medium. The concentration of pimozide was chosen with reference to the clinical dose of 1 mg dissolved in 250 mL of water [28], which corresponds to a concentration of 9 μM. Test chemicals were dissolved in dimethyl sulfoxide (DMSO) and diluted with transport medium (the final DMSO concentration was 1%). Inhibitors, i.e., sertraline (500 μM), aripiprazole (15 μM) and verapamil (100 μM; positive control), were added to the donor side as previously described [24]. These concentrations of sertraline and aripiprazole were chosen in the same manner as described above, based on the calculation that the clinical doses of sertraline (25 mg) and aripiprazole (3 mg) in 250 mL of water would correspond to concentrations of 292 μM and 27 μM, respectively. Samples were collected at 15, 30, 45 and 60 min, and replaced with equal amounts of transport medium. The time course of drug transport in the apical (A) to basal (B) direction (A to B) and that in the opposite direction (B to A) were observed at 37°C. In the transcellular transport studies with Caco-2 cell monolayers, the transport medium on the apical (A) side consisted of HBSS with 10 mM 2-morpholinoethanesulfonic acid (MES) (pH 6.5) and that on the basal (B) side consisted of HBSS with 10 mM HEPES (pH 7.4). Caco-2 cell monolayers were preincubated with the transport medium for 20 min at 37°C. Inhibitor was added to both sides during inhibition studies, and transport experiments were initiated as described above. Samples were collected at 20, 40, 60, 90 and 120 min, and replaced with equal amounts of transport medium.

The permeability (Papp) across cell monolayers was evaluated by dividing the slope of the experimental time course in the A-to-B or B-to-A direction by the concentration on the donor side and is represented as Papp _AtoB_ or Papp _BtoA_, respectively. The efflux ratio was calculated by dividing Papp _BtoA_ by Papp _AtoB_.

### Determination of pimozide

Concentrations of pimozide were determined by LC-MS/MS analysis. Pimozide samples (10 μL) were injected into an HPLC system (LC-20A system, Shimadzu, Kyoto, Japan) equipped with a CAPCELL PAK C18 MGIII/3 μm column (φ2.0 × 50 mm, Shiseido Co. Ltd., Tokyo, Japan). The mobile phase consisted of a mixture of acetonitrile containing 0.1% formic acid (organic solvent phase) and distilled water containing 0.1% formic acid (water phase) (50: 50). The flow rate was 0.1 mL/min, at 40°C. Analytes were detected using a quadrupole mass spectrometer (LCMS-2010EV, Shimadzu) equipped with an electrospray ionization source. Analytes were detected in the positive mode, and the protonated molecular ion of pimozide was monitored at *m/z =* 109.05. The limit of detection was about 0.5 nM.

### Statistical analysis

Kinetic parameters are presented as mean ± standard deviation (S.D.). Other data are presented as mean ± standard error of the mean (S.E.M.). Statistical analysis was performed by means of Steel’s and Dunnet’s test with Pharmaco Basic software (Ver.14.4.2, Scientist, Tokyo). A difference between means was considered to be significant when the *P* value was less than 0.05.

## Results

### ATPase assay for P-gp substrate

**Fig 1:**
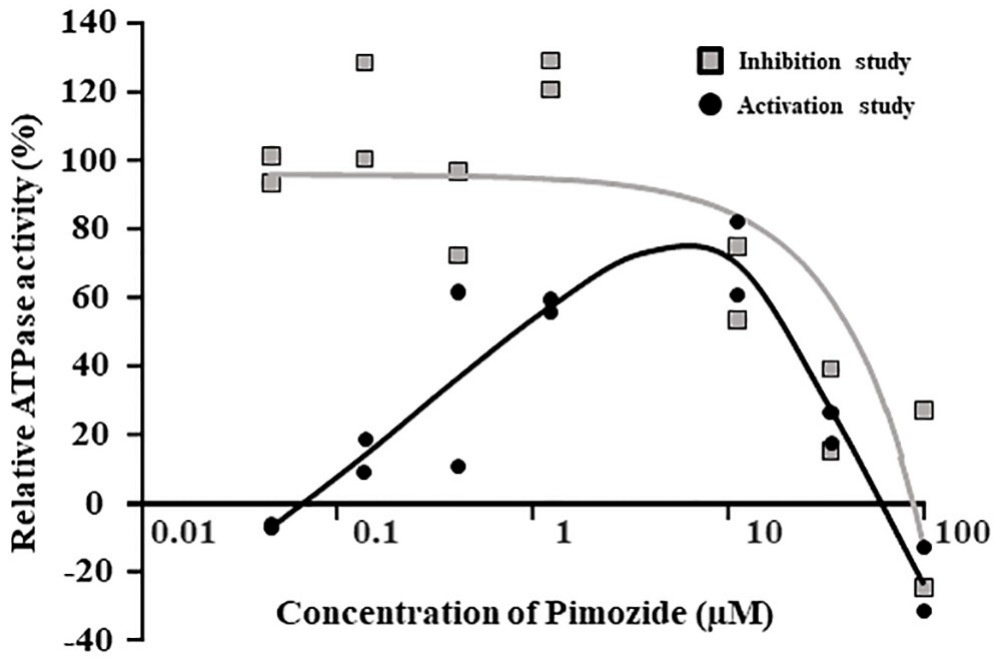
ATPase activity of pimozide. Solid circles show the activation study (n = 2) and open squares show the inhibition study (n = 2). The black line represents the average and the gray line is the fitted curve. Data are given as relative activity (% of control). The pimozide concentration ranged from 0.04 μM to 100 μM.

Concentration-dependent elevation of ATPase activity was initially observed, though concentrations of pimozide over about 10 μM decreased the ATPase activity (Fig 1).

### Efflux and bidirectional assay using P-gp-expressing cells

**Fig 2:**
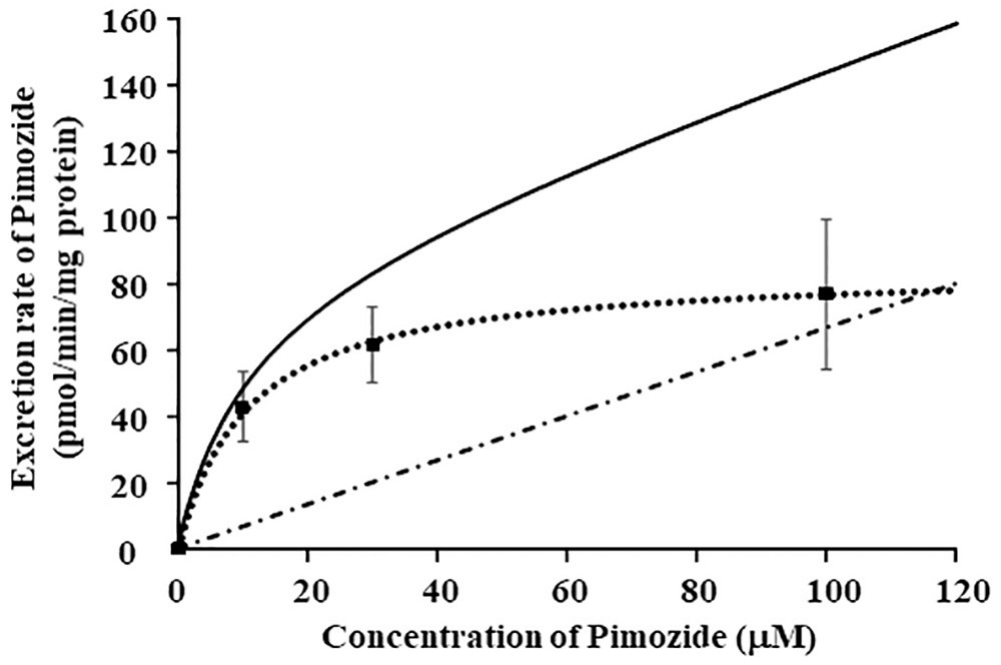
Concentration dependence of pimozide in efflux assay using LLC-GA5-CoL150 cells. The black line represents the efflux rate of pimozide from LLC-GA5-CoL150 cells. The broken lines represent the saturable (∙∙∙∙) and non-saturable (-∙-∙-) transport components. The pimozide concentration ranged from 0.01 μM to 100 μM.

The concentration dependence (in the range of 0.01 to 100 μM) of pimozide efflux from P-gp-expressing LLC-GA5-CoL150 cells is shown in Fig 2. The *J*_max_, *K*_t_ and *k*_d_ values estimated from the efflux assay data according to Eq (1) in Materials and Methods were 84.9 ± 8.9 pmol/min/mg protein, 10.6 ± 4.7 μM and 0.67 ± 0.14 pmol/min/mg protein, respectively. The *J*_max_/*K*_t_ value, i.e., affinity for the transport carrier(s), is 8.0 μL/min/mg protein.

The results of the bidirectional assay using LLC-GA5-CoL150 cell monolayers are shown in Table 1. The efflux ratio of pimozide was 3.62 and that of Rho123 (positive control) was 3.46. Sertraline, aripiprazole and verapamil significantly decreased the efflux ratio to 15.7, 31.4 and 44.6% of the control, respectively.

**Table 1:** Effects of sertraline, aripiprazole and verapamil on pimozide bidirectional assay across LLC-GA5-CoL150 cell monolayers.

	Efflux Ratio	% of control
**Pimozide**	3.62 ± 1.04	100
** + sertraline**	0.57 ± 0.07 *	15.7
** + aripiprazole**	1.14 ± 0.17 *	31.4
** + verapamil**	1.61 ± 0.14 *	44.6
**Rho123**	3.46 ± 0.25	-

The values of the efflux ratio of pimozide in the absence or presence of sertraline, aripiprazole or verapamil in LLC-GA5-CoL150 cell monolayers are shown.

Pimozide: 10 μM, sertraline: 500 μM, aripiprazole: 15 μM, verapamil: 100 μM, Rho123: 10 μM.

Values are mean ± S.E.M. (n = 3~6) and % of control (pimozide alone).

*, *p* < 0.05 compared with pimozide.

### Bidirectional assay across Caco-2 cell monolayers

The Papp of pimozide in the A-to-B direction was indicated 1.64×10^−6^ cm/sec. The Papp in the presence of sertraline was 5.9-fold higher than that in the absence of sertraline. The Papp in the B-to-A direction of pimozide, which was 0.35×10^−6^ cm/sec, was significantly decreased by sertraline, aripiprazole and verapamil. Overall, the addition of sertraline, aripiprazole and verapamil decreased the efflux ratio to 2.9%, 60.6% and 43.9% respectively, compared with pimozide alone (Table 2).

**Table 2:** Effects of sertraline, aripiprazole and verapamil on pimozide bidirectional assay across Caco-2 cell monolayers.

	Papp _AtoB_ (×10^−6^ cm/sec)	Papp _BtoA_ (×10^−6^ cm/sec)	Efflux Ratio
**Pimozide**	1.64±0.14	0.35±0.01	0.21±0.01
** + Sertraline**	9.72±0.71**	0.06±0.01**	0.01±0.00**
** + Aripiprazole**	2.14±0.13	0.28±0.02**	0.13±0.01**
** + Verapamil**	1.58±0.12	0.15±0.00**	0.09±0.00**
**Rho123**	4.91±0.14	14.48±0.33	2.95±0.07
** + Verapamil**	6.88±0.56	12.14±0.23	1.77±0.03

The permeability of pimozide at the concentration of 10 μM was measured in Caco-2 cell monolayers in the absence or presence of sertraline, aripiprazole or verapamil.

Pimozide: 10 μM, sertraline: 500 μM, aripiprazole: 15 μM, verapamil: 100 μM, Rho123: 10 μM.

Values are mean ± S.E.M. (n = 3).

**, *p* < 0.01 compared with pimozide.

## Discussion

There are several reports suggesting that pimozide is a substrate and/or inhibitor of P-gp [20–22], but the data are insufficient to establish the potential for drug-drug interactions at P-gp in the clinical context. In this work, we quantitatively characterized pimozide as a P-gp substrate and/or inhibitor. We found that pimozide concentration-dependently increased the ATPase activity of P-gp-expressing inverted membrane vesicles (Fig 1). Moreover, the ATPase activity was reduced in the high concentration range, indicating that pimozide is both a P-gp substrate and an inhibitor. The ATPase assay is often used to screen for P-gp substrates, and is useful as a simple and convenient means of screening a large number of drugs. Although we used a validated commercial assay kit, this assay is prone to false positives, so we also performed efflux assay using cells overexpressing P-gp for confirmation. The efflux rate of pimozide from P-gp-overexpressing LLC-GA5-CoL150 cells contained both concentration-dependent saturable and non-saturable linear components (Fig 2). The saturable efflux was considered to be mediated by P-gp, in line with a previous report [23]. The *J*_max_/*K*_t_ value, which reflects affinity for the transport carrier(s), is 8.0 μL/min/mg protein. In the gastrointestinal tract, the effective concentration of pimozide is estimated to be at least 9 μM and its *K*_t_ for P-gp was 10.6 μM. These results confirm that pimozide is an effective P-gp substrate, and indicate that it has the potential for drug-drug interaction in the digestive tract. In the bidirectional assay using LLC-GA5-CoL150 cell monolayers, pimozide was transported in the B to A direction, and sertraline and aripiprazole decreased the efflux ratio of pimozide by about 84.3% and 68.6%, respectively (Table 1). Moreover, we next performed the bidirectional assay with Caco-2 cells, which have been widely used for experimental studies of gastrointestinal permeability. The Papp _AtoB_ of pimozide was significantly increased in the presence of sertraline, while Papp _BtoA_ was decreased by sertraline, aripiprazole and verapamil (Table 2) and these findings are consistent with the results of the bidirectional assay using LLC-GA5-CoL150 cells (Table 1). The membrane permeation of pimozide was increased by the P-gp inhibitors, suggesting that the permeation is mediated at least part by P-gp. Because the experimental concentrations of pimozide, sertraline and aripiprazole were set based upon the clinically used doses, it is likely that P-gp-mediated drug-drug interaction in the gastrointestinal tract would occur in the clinical context when these drugs are co-administered. Using Caco-2 cells, the efflux ratio of pimozide was less than 1, indicating that it is well transported in the A to B direction. Pimozide is extremely lipophilic (LogP: 6.3) [29], and it is likely that passive transport contributes to its membrane permeation. In addition, since pimozide is an anionic molecule, organic anion influx transporters expressed in Caco-2 cells may also contribute to its transport, although there is little evidence regarding this issue. Nevertheless, the P-gp inhibitor increased the membrane permeation of pimozide, suggesting that P-gp does contribute in part to pimozide transport.

In conclusion, our results suggest that elevated pimozide blood levels observed when the drug is administered in combination with sertraline and/or aripiprazole can be explained at least in part by interaction at P-gp. Recently, there have been many reports on transporter-mediated drug-drug interactions [30,31], but only a few reports have described deaths that might have been due to transporter-mediated drug-drug interactions in the gastrointestinal tract. It is important to consider the potential risk of drug hyperabsorption due to drug-drug interactions mediated by efflux transporters in the gastrointestinal tract.
